# Flexible fitting of AlphaFold2-predicted models to cryo-EM density maps using elastic network models: a methodical affirmation

**DOI:** 10.1093/bioadv/vbae181

**Published:** 2024-11-18

**Authors:** Maytha Alshammari, Jing He, Willy Wriggers

**Affiliations:** Department of Computer Science, Old Dominion University, Norfolk, VA 23529, United States; Department of Computer Science, Old Dominion University, Norfolk, VA 23529, United States; Department of Mechanical and Aerospace Engineering, Old Dominion University, Norfolk, VA 23529, United States

## Abstract

**Motivation:**

This study investigates the flexible refinement of AlphaFold2 models against corresponding cryo-electron microscopy (cryo-EM) maps using normal modes derived from elastic network models (ENMs) as basis functions for displacement. AlphaFold2 generally predicts highly accurate structures, but 18 of the 137 models of isolated chains exhibit a TM-score below 0.80. We achieved a significant improvement in four of these deviating structures and used them to systematically optimize the parameters of the ENM motion model.

**Results:**

We successfully refined four AlphaFold2 models with notable discrepancies: lipid-preserved respiratory supercomplex (TM-score increased from 0.52 to 0.69), flagellar L-ring protein (TM-score increased from 0.53 to 0.64), cation diffusion facilitator YiiP (TM-score increased from 0.76 to 0.83), and *Sulfolobus islandicus* pilus (TM-score increased from 0.77 to 0.85). We explored the effect of three different mode ranges (modes 1–9, 7–9, and 1–12), masked or box-cropped density maps, numerical optimization methods, and two similarity measures (Pearson correlation and inner product). The best results were achieved for the widest mode range (modes 1–12), masked maps, inner product, and local Powell optimization. These optimal parameters were implemented in the flexible fitting utility elforge.py in version 1.4 of our Python-based ModeHunter package.

**Availability and implementation:**

https://modehunter.biomachina.org.

## 1 Introduction

Deep learning-based structure prediction can produce remarkably accurate atomic models of proteins from their amino acid sequences in the absence of experimental 3D data. For example, DeepMind’s AlphaFold achieved near-experimental accuracy for 87 of the 92 domain targets with a global distance test (GDT_TS) score above 70 (Jumper *et al.* 2021).

AlphaFold uses multiple sequence alignment to quantify the evolutionary relationship between the target sequence and the multiple sequences of known protein structure templates (Jumper *et al.* 2021). This enables AlphaFold to predict the secondary structure of single protein chains with high accuracy, as multiple sequence alignments reveal conserved regions within sequences. However, the accuracy of global fold prediction depends on the coverage of the structure by representative templates (Alshammari *et al.* 2022a). In general, it is challenging to accurately predict inter-domain and inter-chain configurations (quaternary structure). Therefore, there is a risk of compounding prediction errors from the local scale to the global scale of larger proteins, protein–ligand structures, or multi-protein complexes. Such inaccuracies might explain why drug candidates discovered using AlphaFold2 predicted models differ from candidates discovered with structures solved by X-ray crystallography and cryo-electron microscopy (cryo-EM) (Callaway 2024). Further, as AlphaFold2 predictions are only static snapshots and do not reflect the structural flexibility and conformational heterogeneity of proteins (Nussinov *et al.* 2023), rigid AlphaFold2 predictions were found to be only of mixed utility in protein–ligand docking applications (Wong *et al.* 2022).

These limitations can be addressed by a flexible refinement (Tama *et al.* 2004, López-Blanco and Chacón 2013, Kovacs *et al.* 2018) of AlphaFold2 models against cryo-EM maps. The 3D cryo-EM density maps of larger multi-chain and multi-protein assemblies provide the missing global (tertiary and quaternary) structural context to better match the predicted structures with the experimental observations, thereby improving their accuracy. Using flexibility also allows for necessary induced-fit conformational changes at the contact interfaces between chains and proteins. Our work can therefore complement tools for assembling individual fragments and chains into larger assemblies based on the simultaneous docking of fragments (Birmanns *et al.* 2011, He *et al.* 2022, Zhou *et al.* 2022).

Using physical movements of atoms with molecular mechanical force fields, AlphaFold2 models could be improved by refining against experimental cryo-EM maps as low as 5 Å resolution (Alshammari *et al.* 2022a, Terwilliger *et al.* 2022) and against hybrid (resolution-lowered) maps as low as 6 Å resolution (Alshammari *et al.* 2022b). In addition to the map resolution, we observed that the success of the refinement also depended on factors such as the initial accuracy of the AlphaFold2 prediction (Alshammari *et al.* 2022a,b), the local quality of the experimental cryo-EM map (Alshammari *et al.* 2022a), and the specific motion model used in the refinement (Alshammari *et al.* 2023). While we cannot control the first two factors, we can investigate the effect of the motion model.

Our initial studies using the standard Phenix refinement approach (Alshammari *et al.* 2022a,b) allowed for the independent physical movements of all atoms with a molecular mechanical force field. An unexpected benefit of lowering the resolution was observed for two cases (Alshammari *et al.* 2022b), where AlphaFold2 models got trapped in the local optima of higher-resolution cryo-EM maps from which they were able to escape only when the maps were filtered to lower resolution. In a separate case of a three-helix chain from a lipid-preserved respiratory supercomplex, the refinement melted a misaligned helix [Fig. 3C in Alshammari *et al.* (2022a)]; instead, a collective repositioning of the intact helix would have provided a better fit with the cryo-EM map. These observations suggest that it might be beneficial to consider alternative motion models that favor smoothly varying collective deformations over independent atom movements that can get trapped in local minima. The results also highlight the need for an exhaustive search strategy that might be beneficial for escaping from any local traps. We explore these ideas in the following.

To better escape from local optima, we return here to a well-established low-dimensional set of basis functions for conformational changes, namely the lowest-frequency normal modes of the protein chain. Normal mode analysis (Cui and Bahar 2005) is a well-established approximation of the essential dynamics of biomolecular structures, and it has been used for more than two decades in the flexible fitting of structures to cryo-EM maps (Chacon *et al.* 2003, Tama *et al.* 2004, Zheng 2011, Tekpinar 2018, Krieger *et al.* 2022, Vuillemot *et al.* 2022). In a recent proof of concept, we implemented a coarse-grained elastic network model (ENM) to generate an exhaustive set of structure decoys from discretely sampled modes (Alshammari *et al.* 2023). ENMs reduce the number of degrees of freedom of motion (compared to three times the number of atoms in an independent atom model) by using only the lowest-frequency normal modes (Tama *et al.* 2004, López-Blanco and Chacón 2013), yielding a much more manageable (lower-dimensional) representation of conformational space (at the cost of ignoring local high spatial-frequency deformations). Our results demonstrate a notable improvement in the accuracy of the three-helix chain of the lipid-preserving mitochondrial respiratory supercomplex, where one of the decoys exhibited a TM-score of 0.68 compared to the AlphaFold2 TM-score of 0.52 (Alshammari *et al.* 2023). This success reduces the flexible refinement problem to the problem of picking the correct decoy. For the present work, we implemented a new tool, elforge.py, in ModeHunter version 1.4, which optimizes a continuously varying decoy by matching it with the cryo-EM map, similar to other fitting tools (Tama *et al.* 2004, López-Blanco and Chacón 2013).

The implementation of flexible refinement in a Python-based package is particularly timely because it allows us to rapidly explore and prototype several local and global optimization strategies that are readily available within standard Python libraries. In our study, we utilized SciPy (Virtanen *et al.* 2020), an open-source Python library that provides a wide range of tools and algorithms, including optimization, linear algebra, and interpolation, among other computational tasks. We tested the performance of the following four optimization techniques from SciPy: Powell, Nelder–Mead, Dual Annealing, and Differential Evolution. Powell is a local optimization method that efficiently searches for the nearest local minimum of a function without requiring calculation of the derivative. It adjusts its internal parameter iteratively by exploring conjugate directions that speed up convergence (Powell 2024). Nelder–Mead is another derivative-free optimization method (Nelder–Mead 2024). This technique iteratively shrinks and reshapes an *n*-dimensional simplex into coverage toward the minimum of the function. The simplex is a geometric shape with *n *+* *1 points in *n*-dimensional space, where *n* represents the number of variables being optimized. Nelder–Mead evaluates the function at each simplex point and replaces the farthest point from the minimum with a point closer to the estimated minimum. In contrast, Differential Evolution is a population-based stochastic global optimization method that maintains a population of candidate solutions and iteratively creates new candidate solutions by introducing variations based on differences in existing solutions to minimize the objective function (Differential Evolution 2024). Dual Annealing, another global optimization method implemented in SciPy, is a variant of fast simulated annealing (FSA) (Szu and Hartley 1987). The simulated annealing method was inspired by metallurgical annealing using a cooling schedule (Bertsimas and Tsitsiklis 1993). Starting at a high temperature, the method explores the search space randomly and gradually moves to a more deterministic (local) exploration as the temperature decreases. FSA adjusts the temperature dynamically based on the optimization process, resulting in faster convergence toward the global minimum (Szu and Hartley 1987). Dual annealing is built upon FSA and combines it with a local search for convergence efficiency (Dual Annealing 2024).

Another question we addressed in the present study is how to derive the greatest benefit from the computer run time invested, since there are tradeoffs between the different methods. Typically, local optimization is much more efficient than global optimization. We explored the question of whether global optimization is worth the extra time investment or whether a larger basis set of normal modes would yield better benefits for the types of cases selected in this study.

The selection of normal modes requires careful validation. In the composition of multiple modes, each mode contributes one degree of freedom (or dimension) to the dynamical model. The first six normal modes (when ordered by frequency) are rigid-body motions (linear combinations of translation and rotation). These modes are special because they are not resisted by the springs of the ENM (Section 2.2) and have zero frequency. These rigid-body modes cannot be numerically resolved into individual transitions or rotations and need to be added as a complete subset to the basis set. We ask whether it is worth including these first six modes in the refinement of the AlphaFold2 model or whether only the internal deformations should be used starting from the first “non-trivial” (resisted by ENM springs, frequency >0) mode number 7. Further, once the start index of the mode range is established, what should be the highest frequency mode included in the basis set? Due to the “curse of dimensionality” (Altman and Krzywinski 2018), an exhaustive exploration becomes increasingly difficult, resulting in a tradeoff between computing efficiency and accuracy that needs to be established empirically.

The objective function we minimize in our flexible refinement is the negative correlation coefficient (CC) between the simulated map (obtained from the deformed and resolution-lowered AlphaFold2 model) and the target cryo-EM map. Deformation involves systematically adjusting the mode elongation coefficients, which are the coordinates of the deformation within the selected basis of normal modes. The aim is to find an optimum combination of the mode elongation vector to generate a structure that best fits the target cryo-EM map as measured by maximizing the CC. In the present work, we used two types of target density maps: masked and box-cropped maps. The masked maps exclude the irrelevant (unaccounted for) density regions, resulting in simpler refinement of a protein chain (as is typically done in many flexible fitting validation studies). By contrast, box-cropped maps are more often encountered in practical applications and provide a bigger challenge for the fitting due to the extra density from neighboring chains, leading to false positives and spurious local optima.

An additional complication requiring validation is the possibility that the deformed structure extends beyond the target map boundaries. Simple cropping of any extraneous atoms would affect the Pearson product-moment CC value, which centers the data by subtracting the average densities (see below). This would introduce an unwanted cropping bias in our optimization. To reduce this bias, we implemented a zero-padding of the target map (by adding a margin of zeros at each exposed face of the target volume). This margin cannot be adjusted on the fly because the Pearson CC is not invariant under zero-padding. Further, the extra margin of zeros reduces computational efficiency; thus, it cannot be set arbitrarily large. To avoid this problem, we also employed an alternative uncentered CC implemented in our Situs package [Section 6 in Wriggers (2012)]. This CC value (essentially a normalized inner product) is invariant under zero-padding and does not introduce a cropping bias. It uses uncentered data, which is appropriate for non-negative images; however, without data centering, it may be less discriminative than the Pearson CC [Section 6 in Wriggers (2012)]. In the next section, we compare Pearson CC with a fixed margin and the inner product.

## 2 Methods

A test set of four protein chains was employed to assess the performance of the flexible fitting method (Section 2.1). Normal modes with ENM were developed with a new ModeHunter version 1.4 (Stember and Wriggers 2009) (Section 2.2) and utilized for flexibly fitted Alphafold2 predicted models (Section 2.3) into Cryo-EM for refinement.

### 2.1 Data preparation and structure prediction with AlphaFold2

Four test cases were selected from a full set of 137 AlphaFold2 predictions. Overall, 87% of the AlphaFold2 predictions already matched the expected structures very well. Only 18 of the 137 cases exhibited a TM-score below 0.80, suggesting that these candidates (second column of Supplementary Table S1) could benefit from flexible refinement against a cryo-EM map. From these candidates, four promising cases were ultimately selected after decoy generation (see Supplementary Data). The four test systems encompassed an amino acid sequence for AlphaFold2 prediction, a known atomic structure for validation purposes, and a cryo-EM density map for the flexible fitting of the AlphaFold2 models:

*Lipid-preserved respiratory supercomplex*: The atomic structure 7DGQ chain 3 was obtained from the Protein Data Bank (PDB) in June 2022. The corresponding EMD 30673 (resolution 5 Å) was obtained from the Electron Microscopy Data Bank (EMDB).

*Flagellar L-ring protein*: The Free Modeling case at CASP14 T1047s1-D1 was accompanied by a cryo-EM map (EMD 12183), from which a hybrid map with a resolution of 5 Å was created (Alshammari *et al.* 2022b). The corresponding atomic structure, 7BGL chain A, was downloaded from the PDB in March 2022.

*Cation diffusion facilitator YiiP*: A hybrid map with a resolution of 5 Å was created from EMD 23093 (Alshammari *et al.* 2022b). The PDB entry 7KZZ chain B was obtained from the depository of Terwilliger *et al.* (2022), representing data downloaded in August 2021.

*Sulfolobus islandicus pilus*: The atomic structure of 6NAV chain A was obtained from the PDB in March 2023. The corresponding EMD 0397 (resolution 4.1 Å) was obtained from the Electron Microscopy Data Bank.

We generated AlphaFold2 models using the Google Cloud platform Colab for the first three test systems, as described by Terwilliger *et al.* (2022), AlphaFold with a density map (2022), Alshammari *et al.* (2022a,b). For the last system, the AlphaFold2 model was generated using the Phenix software utility “predict_and_build” (Adams *et al.* 2010).

The web interface in Colab (AlphaFold with a density map 2022) and the Phenix software utility “predict_and_build” (Adams *et al.* 2010) generate a pure AlphaFold2 model and a refined AlphaFold2 model. These methods start with structure prediction using AlphaFold2 and then refine the models using Phenix (Terwilliger *et al.* 2022). In our study, we utilized only the pure AlphaFold2 model. The AlphaFold2 model was then aligned with the true structure using TM-align (Zhang and Skolnick 2005).

The density maps were rectangular cropped to contain the chain of interest *via* the Phenix tool phenix.map_box (Adams *et al.* 2010). In the first three cases from our previous studies (see Supplementary Data), maps were also filtered using the Phenix tool phenix.local_aniso_sharpen, consistent with Alshammari *et al.* (2022a,b). All maps were masked using a UCSF Chimera command: “vop zone #0 #1 5 minimalBounds true modelID #2” (Pettersen *et al.* 2004). Here, #0 represents the cryo-EM map, while #1 denotes the true structure. The values of grid points beyond a radius of 5 Å from any atom specified in the atom-spec were set to zero, effectively masking those areas beyond the defined zone. The parameter “minimalBounds” ensured that the resulting map was compact while enclosing the specified zone, with #2 assigned as the masked map ID. Subsequently, the box-cropped region and masked map were converted from MRC to Situs format using the map2map utility in the Situs package (Wriggers 2012). Other than described in this section, the true structure was used only for validation purposes (final TM-score calculation) in Section 3.

### 2.2 Normal mode generation with elastic network models

ENMs of proteins comprise carbon-alpha atoms interconnected by linear (Hookean) springs within a defined cutoff distance. When utilized to represent protein dynamics, the normal modes of ENMs yield a reduced-dimensional set of basis functions that can be used to generate oscillating motions. The ModeHunter (Stember and Wriggers 2009) utility enmhunt.py was applied to generate normal modes and frequencies with the recommended cutoff distance of 12 Å. The Hessian matrix, representing the second derivatives of the pairwise harmonic potential energy function, was diagonalized to extract the eigenvectors and eigenvalues corresponding to the normal modes and frequencies, respectively. Subsequently, the computed modes and frequencies were saved in Python pickle files, and the ModeHunter tool augment.py was utilized to interpolate the modes to all atoms.

### 2.3 Flexible fitting of AlphaFold2 models into cryo-EM maps

To flexibly fit the AlphaFold2 models into the density map, the new elforge.py tool was developed for ModeHunter version 1.4. The method generates fitted structures in PDB format using eight parameters: the PDB name of the AlphaFold2 model (Section 2.1), the density map (Section 2.1), the resolution of the density map, the pickle file that contains the all-atom modes (Section 2.2), the start and end index of the mode range (Section 1), the zero-padding margin, and the output name. The margin parameter describes the zero-padding in voxel units added around the simulated density map of the deformed structure and is required for large movements of the structure during fitting (Section 1) when using Pearson CC optimization.

The flexible fitting method begins by loading the required data, including modes and frequencies, and reading the input AlphaFold2 structure alongside the volumetric target map. Then, the SciPy function “scipy.optimize” is employed to conduct the flexible fitting process. This involves optimizing the similarity measure to adjust the coefficients of the linear combination of normal modes (elongation vector) applied to the AlphaFold2 atomic structure until it aligns with the target EM map. During this process, the molecular structure undergoes flexible adjustments based on the mode elongation vector (Section 1). The final deformed structure is then mapped onto a voxel grid, convolved with a Gaussian kernel (to match the resolution of the target cryo-EM map according to the Situs convention) (Wriggers 2012), and evaluated for CC with the target map.

For the optimization phase, the mode range is set based on specified start and end indices (Section 1). The negative CC serves as the objective function to be minimized, and the chosen SciPy optimizer (Powell, Nelder–Mead, Dual Annealing, or Differential Evolution, see Section 1) is utilized to determine the optimal mode elongation vector. The initial guess for the elongation vector was set to 0 in Powell and Nelder–Mead, while the bound parameters in Dual Annealing and Differential Evolution were set to −9 (lower bound) and +9 (upper bound) to bracket the observed range of elongations during Powell and Nelder–Mead optimizations.

The Python NumPy library was employed to compute the CC between the target map and the simulated map of the deformed structure during the fitting process. In this article, we explored two different CC measures to quantify similarity. CC measures are widely used in cryo-EM fitting for this purpose (Vasishtan and Topf 2011, Wriggers 2012, Maurer *et al.* 2024). The calculation of the Pearson product-moment correlation coefficient was conducted using the NumPy method “numpy.corrcoef”. The Pearson CC between two densities *x* and *y* over *n* voxels is defined as $\mathrm{CC}=\sum_{i=1}^{n}\left(x_{i}-\bar{x}\right)\left(y_{i}-\bar{y}\right)/(\sqrt{\sum_{i=1}^{n}{\left(x_{i}-\bar{x}\right)}^{2}}\sqrt{\sum_{i=1}^{n}{\left(y_{i}-\bar{y}\right)}^{2}})$, where $\bar{x}\;$and $\bar{y}$ are the average densities. This method centers the data by subtracting the averages; thus, it is sensitive to zero-padding of the map and ideally requires a fixed margin of zeros that is large enough to embed all flexibly fitted intermediate conformations to avoid any biases of the map boundary on the fitting. (Here, the margin parameter was set to 10 voxels, where the Pearson CC was used.) Another similarity measure method, “numpy.inner”, was employed to compute the inner product of the simulated and target map: $\mathrm{CC}=\sum_{i=1}^{n}\left(x_{i}\right)\left(y_{i}\right)$. This measure is similar to the CC implementation in Situs: $\mathrm{CC}=\sum_{i=1}^{n}\left(x_{i}\right)\left(y_{i}\right)/\left(\sqrt{\sum_{i=1}^{n}{\left(x_{i}\right)}^{2}}\sqrt{\sum_{i=1}^{n}{\left(y_{i}\right)}^{2}}\right)$ [see Section 6 in Wriggers (2012)]. Both methods utilize uncentered maps; however, unlike in Situs, the inner product here was not normalized to save computing time. The use of uncentered densities in the inner product allowed us to run the flexible fitting without a margin of zeroes, since the CC values are invariant under zero-padding of the map (so there are no cropping biases in the flexible fitting, and any part of the deformed structures that are protruding from the map can simply be ignored). It is also noteworthy that the cross-correlation value was multiplied by −1 because the optimization functions in SciPy all aim to minimize the objective function, whereas our aim is to maximize the CC values.

For each of the four test systems (Section 2.1), flexible fitting was assessed using two types of cryo-EM maps: box-cropped and masked (see Sections 1 and 2.1). Three different mode ranges (Section 1) were tested: 1–9, 7–9, and 1–12. Upon identification of the optimal elongation vector, atomic coordinates were updated accordingly, and a PDB file was saved to store the flexibly fitted structure. TM-align (Zhang and Skolnick 2005) was used to quantify the accuracy of the AlphaFold2 model before and after flexible fitting. We used TM-align with default parameters (sequence-independent) in the main text below. Sequence-dependent TM-scores were substantially the same (see Supplementary Data).

## 3 Results

We systematically examined four AlphaFold2 test system discrepancies using ENM-based flexible fitting. Each AlphaFold2 model (screened from a larger set, see Section 2.1) and flexibly fitted model in this article were aligned with the true structure using TM-align, and the TM-score, ranging from 0 to 1, was used to evaluate the quality of the models (Zhang and Skolnick 2005). TM-align is a protein structural comparison algorithm that is sequence-independent (when used with default parameters, as implemented in this and in our earlier papers), employing heuristic dynamic programming iterations to ascertain the optimal superposition between the two structures (Zhang and Skolnick 2005). To find the best fitting of AlphaFold2 models into the Cryo-EM density maps, we employed a comprehensive search strategy utilizing various optimization methods (Powell, Nelder–Mead, Dual Annealing, and Differential Evolution), across a range of fitting parameters, including the normal modes (1–9, 7–9, 1–12), two types of density map (box-cropped and masked), and two similarity measures (Pearson CC and inner product). We begin by graphically illustrating the best results in figures before describing detailed results of all calculations provided in tables.

### 3.1 Visual structure inspection

**Figure 1.**
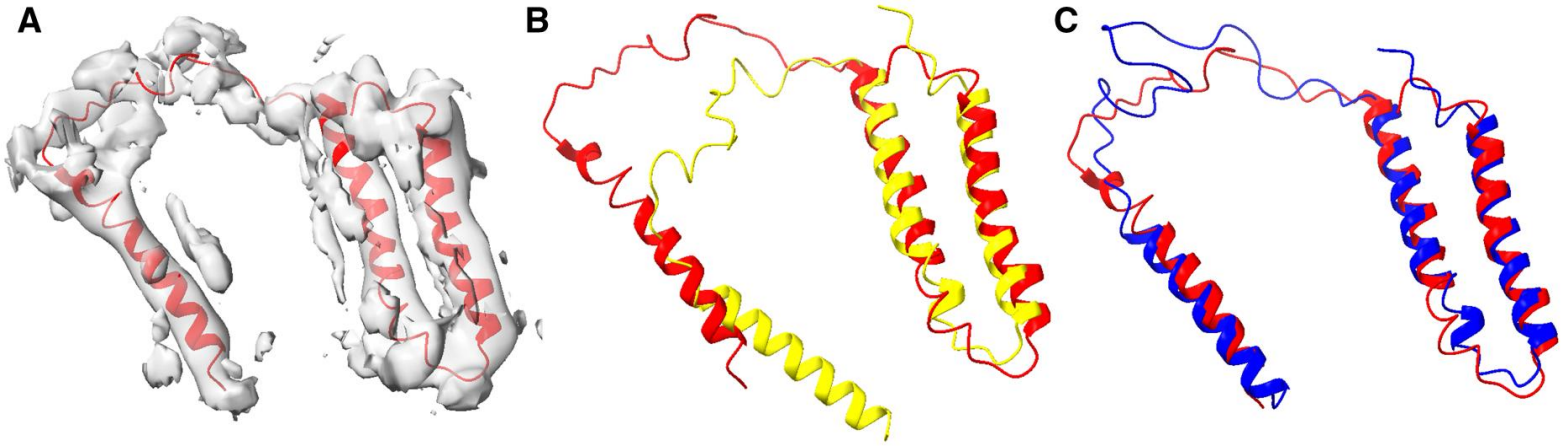
Lipid-preserved respiratory supercomplex models obtained from AlphaFold2 prediction and flexible fitting. (A) The true protein structure (red, chain 3 of PDB ID 7DGQ) and its associated 5 Å resolution masked density map (EMD 30673). (B) Superposition of the true structure (red) and the model obtained from AlphaFold2 (yellow). (C) AlphaFold2 model (blue) flexibly fitted to the density map aligned with the true structure (red). The parameters used in the flexible fitting were masked map, Pearson, modes 1–12, and Powell. The superposition was conducted utilizing TM-align, yielding TM-scores of 0.52 and 0.69 in panels B and C, respectively. The molecular graphics shown in Figures 1–4 were generated using ChimeraX (Pettersen et al. 2021).

The lowest initial accuracy of the AlphaFold2 predicted models in our test cases exhibited a TM-score of 0.52 when compared to the true chain 3 of PDB ID 7DGQ (Fig. 1). This chain is part of a lipid-preserved mitochondrial respiratory supercomplex. The chain has a length of 115 residues and corresponds to the 5 Å cryo-EM map 30673 (Fig. 1A). Whereas the existence of three helices was accurately predicted by AlphaFold2, the relative positioning of the third helix was not accurate (Fig. 1B). When flexible fitting was applied (parameters: see the caption of Fig. 1), a significant improvement was observed, particularly in the third helix and its connecting loop (Fig. 1C). This also led to a notable increase in the TM-score from 0.52 to 0.69 (Table 1, column 6). The refinement success demonstrates the utility of the ENM-based flexible fitting approach, especially in situations where deformation is global and relatively smooth across the entire structure.

**Table 1.** Accuracy of models before and after flexible fitting using the Powell optimization method.

System^a^	Res. (Å)^b^	AF2 TM^c^	Masked map—aligned AF2^d^						Boxed map—aligned AF2^e^					
			Pearson—margin 10			Inner product			Pearson—margin 10			Inner product		
			Modes 1–9	Modes 7–9	Modes 1–12	Modes 1–9	Modes 7–9	Modes 1–12	Modes 1–9	Modes 7–9	Modes 1–12	Modes 1–9	Modes 7–9	Modes 1–12
1	5	0.52	0.64	0.58	0.69	0.62	0.57	0.68	0.60	0.58	0.52	0.60	0.58	0.53
2	5	0.53	0.63	0.59	0.59	0.63	0.58	0.64	0.61	0.59	0.59	0.61	0.59	0.61
3	5	0.76	0.80	0.80	0.83	0.80	0.80	0.83	0.80	0.80	0.83	0.80	0.80	0.83
4	4.10	0.77	0.75	0.76	0.79	0.78	0.77	0.85	0.76	0.76	0.77	0.77	0.77	0.81

a Test cases. See section 2.1 for details.

b Resolution of the map.

c TM-score of the AF2-predicted model.

d TM-score of the model fitted to the masked map.

e TM-score of the model fitted to the boxed map.

**Figure 2.**
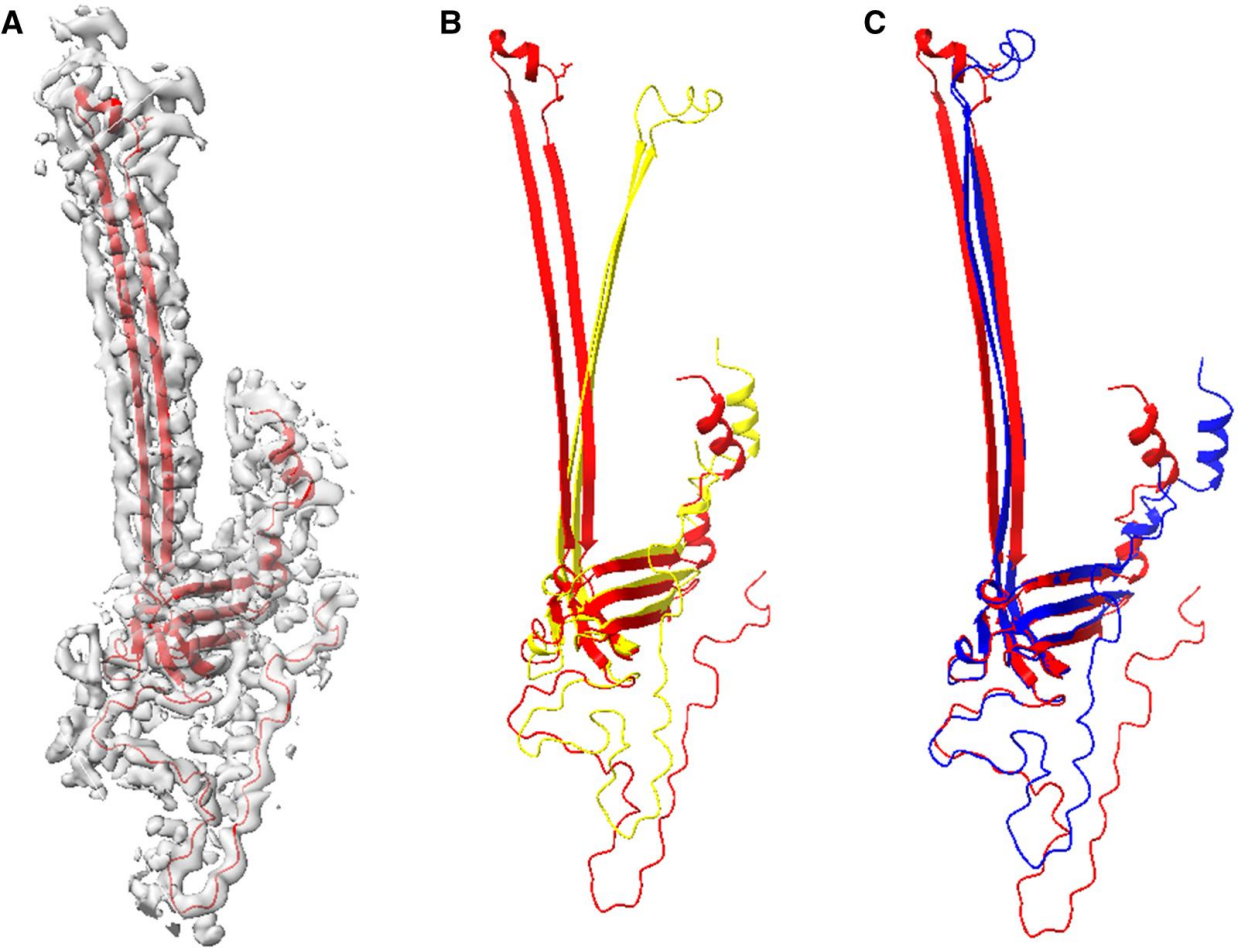
Flagellar L-ring protein models obtained from AlphaFold2 prediction and flexible fitting. (A) The true protein structure (red, chain A of PDB ID 7BGL) and its associated 5 Å resolution masked hybrid map derived from EMD 12183. (B) Superposition of the true structure (red) and the model obtained from AlphaFold2 (yellow). (C) AlphaFold2 model (blue) flexibly fitted to the density map aligned with the true structure (red). The parameters used in the flexible fitting were masked map, inner product, modes 1–9, and Powell. The superposition was conducted utilizing TM-align, yielding TM-scores of 0.53 and 0.63 in panels B and C, respectively.

In the second test system, the flagellar L-ring protein (Fig. 2), the AlphaFold2 model had an initial low TM-score of 0.53 (Fig. 2B). This chain was part of a challenging Free Modeling category in CASP14, known for its difficult target structures. Despite the overall correctness of the fold and well-predicted secondary structures, discrepancies in the relative positioning of a long sheet were noted. We employed the ENM-based flexible refinement against a hybrid 5 Å map (Fig. 2A; parameters in the caption) to address these inaccuracies, resulting in a significant enhancement in the alignment and fitting of the AlphaFold2 predicted model with the true chain A of PDB ID 7BGL, particularly in the region of interest corresponding to the long sheet (Fig. 2C). The TM-score also improved notably from 0.53 to 0.63 (Table 1, column 7), demonstrating a better alignment with the true structure.

**Figure 3.**
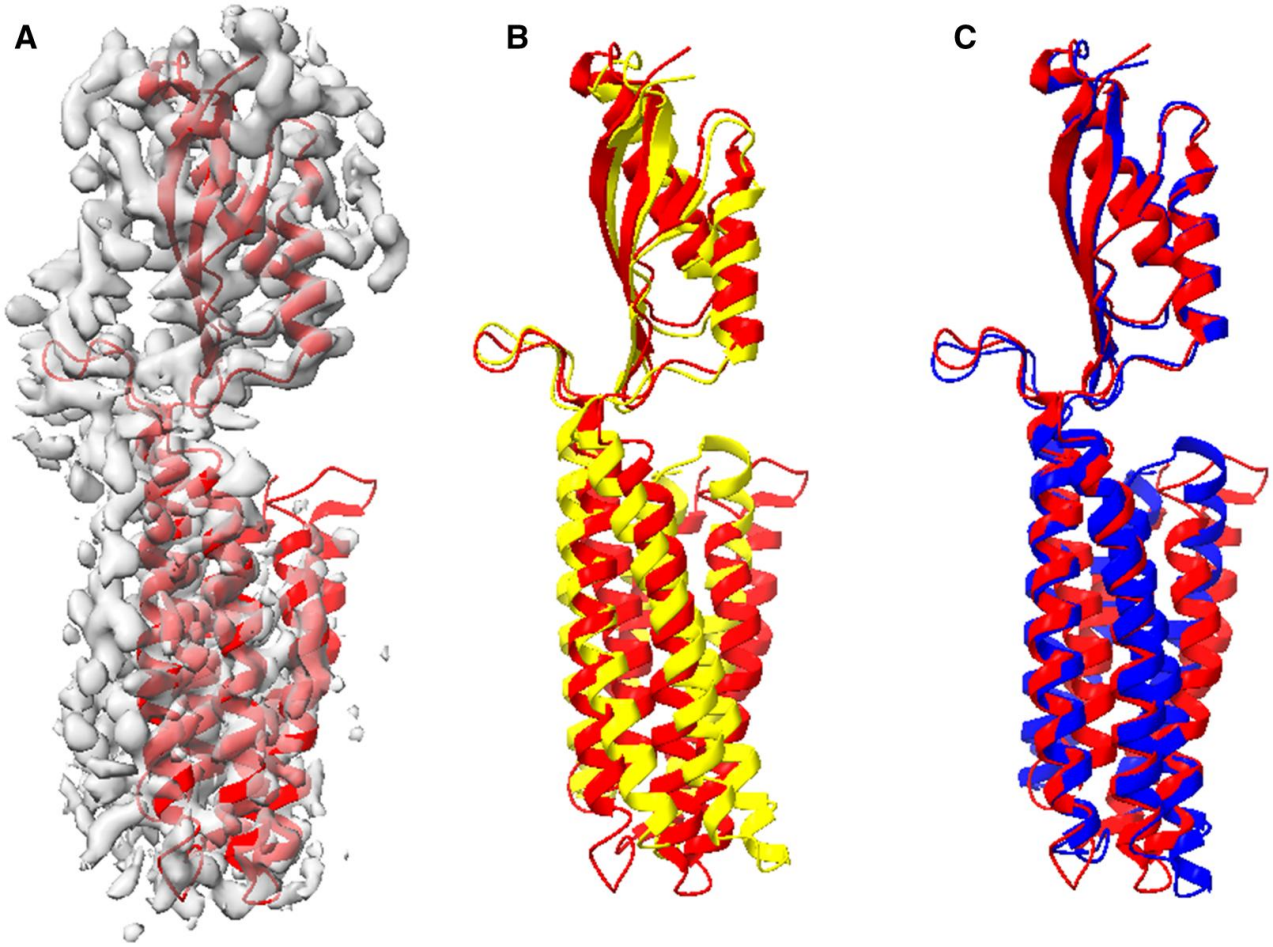
Cation diffusion facilitator YiiP models obtained from AlphaFold2 prediction and flexible fitting. (A) The true protein structure (red, chain B of PDB ID 7KZZ) and its associated 5 Å resolution masked hybrid map derived from EMD 23093. (B) Superposition of the true structure (red) and the model obtained from AlphaFold2 (yellow). (C) AlphaFold2 model (blue) flexibly fitted to the density map aligned with the true structure (red). The parameters used in the flexible fitting were masked map, inner product, modes 1–12, and Powell. The superposition was conducted utilizing TM-align, yielding TM-scores of 0.76 and 0.83 in panels B and C, respectively.

In contrast to the previous systems, two of our test systems showed higher initial TM-scores above 0.70 for the AlphaFold2 predicted models, specifically in cation diffusion facilitator YiiP (Fig. 3). Whereas the upper domain was accurately predicted by AlphaFold2, discrepancies in the lower domain affected the overall structure prediction accuracy (Fig. 3B). Flexible fitting to a hybrid 5 Å map (Fig. 3A) reduced these discrepancies (Fig. 3C), resulting in a significant improvement in the TM-score from 0.76 to 0.83 (Table 1, column 9).

**Figure 4.**
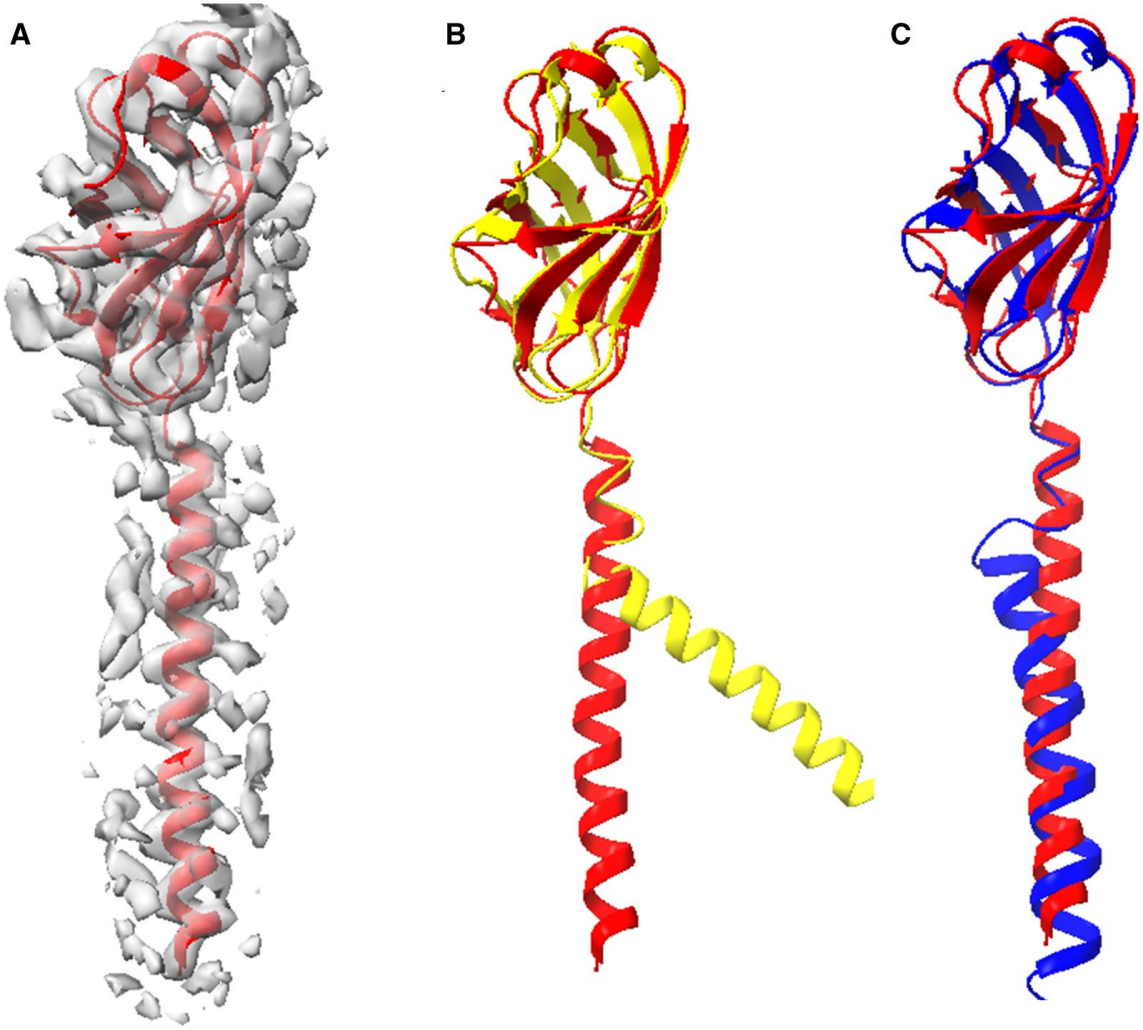
*Sulfolobus islandicus* pilus models obtained from AlphaFold2 prediction and flexible fitting. (A) The true protein structure (red, chain A of PDB ID 6NAV) and its associated 4.10 Å resolution masked density map (EMD 0397). (B) Superposition of the true structure (red) and the model obtained from AlphaFold2 (yellow). (C) AlphaFold2 model (blue) flexibly fitted to the density map aligned with the true structure (red). The parameters used in the flexible fitting were masked map, inner product, modes 1–12, and Powell. The superposition was conducted utilizing TM-align, yielding TM-scores of 0.77 and 0.85 in panels B and C, respectively.

The final test system focused on a *S. islandicus* pilus (Fig. 4), which was associated with an experimental density map of resolution 4.1 Å (Fig. 4A). Similar to the previous system, this chain comprised an upper and lower domain and an initial TM-score above 0.70. While the upper domain was accurately predicted by AlphaFold2, the lower domain helix (Fig. 4B) exhibited a kink that was not observed in the true structure. The flexible fitting attempted to straighten the helix, resulting in a notable improvement in the TM-score from 0.77 to 0.85 (Table 1, column 9). However, the relatively local and sharp kink could not be completely repaired by the ENM-based refinement, which uses smoothly varying (global) low-frequency normal modes (Fig. 4C). This is because ENMs cannot form or break contacts between side chains, which would require alternative modeling approaches.

### 3.2 Effect of map masking

The results generally indicated that when masked maps were used for flexible fitting, the model’s accuracy significantly improved compared to models refined with box-cropped (unmasked) maps. For example, as shown in Table 1 for the first system, the TM-score improved when a masked map was used (with Pearson CC and various modes ranges), increasing from 0.52 to 0.64, 0.58, and 0.69 (Table 1, columns 4–6). The corresponding TM-scores of the box-cropped map were 0.60, 0.58, and 0.52, respectively (Table 1, columns 10–12), indicating that the extraneous densities from neighboring chains can reduce the effectiveness of the refinement. Similarly, for the fourth system, the TM-score of the AlphaFold2 model was 0.77. The highest TM-score for refinement against the masked map was 0.85 (Table 1, column 9; for inner product and mode range 1–12), but the corresponding score refined against the box-cropped map was only 0.81 (Table 1, column 15). This example suggests that unmasked maps can still be useful, albeit to a lesser degree, compared to masked maps.

### 3.3 Effect of wider mode range

The results clearly showed that the refined models exhibited higher accuracy when incorporating a wider range of modes, including rigid-body modes (1–6) and higher-frequency modes (10–12). Although using a larger number of modes increases the dimensionality of the optimization, the investment of more computational resources was warranted in this case. For example, in the last system of Table 1, it was challenging to enhance the accuracy of its AlphaFold2 model (TM-score 0.77) when only low-frequency modes 1–9 or 7–9 (no rigid-body fitting) were used, even when refining against the masked map (TM-scores between 0.75 and 0.78). However, the TM-score significantly increased to 0.83 (Table 1, column 9) when a wider mode range of 1–12 was used in this case. Similarly, when the box-cropped map was utilized in the fitting, the accuracy improved to 0.80, which still indicates a significant improvement and justifies using a wide range of modes (Table 1, column 15).

### 3.4 Effect of CC and zero-padding margin

Comparing Pearson CC (with a margin of 10 voxels) and our more efficient and padding invariant inner product, the results in Tables 1–4 show that refined models with the inner product yield equal or better accuracy than Pearson CC in 77 of the 96 cases, with the remaining 19 cases exhibiting very similar scores. Note that when box-cropped maps and Powell optimization were used, we observed in all inner product cases an equal or higher TM-score than with Pearson CC (Table 1, columns 13–15).

**Table 2.** Accuracy of models before and after flexible fitting using the Nelder–Mead optimization method.

System^a^	Res. (Å)^b^	AF2 TM^c^	Masked map—aligned AF2^d^						Boxed map—aligned AF2^e^					
			Pearson—margin 10			Inner product			Pearson—margin 10			Inner product		
			Modes 1–9	Modes 7–9	Modes 1–12	Modes 1–9	Modes 7–9	Modes 1–12	Modes 1–9	Modes 7–9	Modes 1–12	Modes 1–9	Modes 7–9	Modes 1–12
1	5	0.52	0.56	0.58	0.56	0.57	0.57	0.56	0.56	0.58	0.58	0.58	0.58	0.58
2	5	0.53	0.59	0.59	0.59	0.58	0.58	0.59	0.56	0.59	0.58	0.58	0.59	0.57
3	5	0.76	0.80	0.80	0.82	0.79	0.80	0.82	0.80	0.80	0.82	0.79	0.80	0.81
4	4.10	0.77	0.77	0.76	0.79	0.77	0.77	0.79	0.76	0.76	0.79	0.78	0.77	0.78

a Test cases. See section 2.1 for details.

b Resolution of the map.

c TM-score of the AF2-predicted model.

d TM-score of the model fitted to the masked map.

e TM-score of the model fitted to the boxed map.

**Table 3.** Accuracy of models before and after flexible fitting using the Dual Annealing optimization method.

System^a^	Res. (Å)^b^	AF2 TM^c^	Masked map—aligned AF2^d^						Boxed map—aligned AF2^e^					
			Pearson—margin 10			Inner product			Pearson—margin 10			Inner product		
			Modes 1–9	Modes 7–9	Modes 1–12	Modes 1–9	Modes 7–9	Modes 1–12	Modes 1–9	Modes 7–9	Modes 1–12	Modes 1–9	Modes 7–9	Modes 1–12
1	5	0.52	0.56	0.58	0.54	0.65	0.57	0.51	0.36	0.58	0.25	0.53	0.58	0.50
2	5	0.53	0.63	0.59	0.61	0.63	0.58	0.64	0.58	0.59	0.60	0.59	0.59	0.56
3	5	0.76	0.80	0.80	0.52	0.80	0.80	0.63	0.58	0.80	0.48	0.77	0.80	0.69
4	4.10	0.77	0.85	0.76	0.85	0.85	0.77	0.85	0.40	0.76	0.36	0.85	0.77	0.72

a Test cases. See section 2.1 for details.

b Resolution of the map.

c TM-score of the AF2-predicted model.

d TM-score of the model fitted to the masked map.

e TM-score of the model fitted to the boxed map.

**Table 4.** Accuracy of models before and after flexible fitting using the Differential Evolution optimization method.

System^a^	Res. (Å)^b^	AF2 TM^c^	Masked map—aligned AF2^d^						Boxed map—aligned AF2^e^					
			Pearson—margin 10			Inner product			Pearson—margin 10			Inner product		
			Modes 1–9	Modes 7–9	Modes 1–12	Modes 1–9	Modes 7–9	Modes 1–12	Modes 1–9	Modes 7–9	Modes 1–12	Modes 1–9	Modes 7–9	Modes 1–12
1	5	0.52	0.64	0.58	0.69	0.65	0.57	0.51	0.42	0.58	0.19	0.57	0.58	0.45
2	5	0.53	0.62	0.59	0.61	0.63	0.59	0.62	0.61	0.59	0.60	0.61	0.59	0.59
3	5	0.76	0.80	0.80	0.83	0.80	0.80	0.83	0.80	0.80	0.49	0.80	0.80	0.83
4	4.10	0.77	0.85	0.76	0.81	0.85	0.77	0.85	0.28	0.76	0.30	0.85	0.77	0.72

a Test cases. See section 2.1 for details.

b Resolution of the map.

c TM-score of the AF2-predicted model.

d TM-score of the model fitted to the masked map.

e TM-score of the model fitted to the boxed map.

### 3.5 Effect of optimization methods

Finally, we compared the refinement results achieved by two local optimization methods, Powell and Nelder–Mead, and two global optimization methods, Dual Annealing and Differential Evolution. The total run times on a single Intel Xeon CPU E5-2600 series were 6 h 41 m (Table 1, Powell), 10 h 52 m (Table 2, Nelder–Mead), 52 h 54 m (Table 3, Dual Annealing), and 67 h 17 m (Table 4, Differential Evolution). Our results showed that the slow global optimization methods offered no real benefit over the more efficient local optimization methods, such as Powell. The first system exhibited an optimum refinement (TM-score increased from 0.52 to 0.69) with both Powell and Differential Evolution (column 6 in Tables 1 and 4). The second system exhibited an optimum refinement (TM-score increased from 0.53 to 0.64) with both Powell and Dual Annealing (column 9 of Tables 1 and 3). The third system exhibited an optimum refinement (TM-score increased from 0.76 to 0.83) with both Powell and Differential Evolution (column 9 of Tables 1 and 4). Moreover, the fourth system exhibited an optimum refinement (TM-score increased from 0.77 to 0.85) with Powell, Dual Annealing, and Differential Evolution (column 9 of Tables 1, 3, and 4). In all four systems, the efficient Powell method was therefore the most prudent choice. Moreover, the results showed that the expensive global optimization methods may actually severely diminish refinement performance in the case of the more challenging box-cropped maps, probably due to the “curse of dimensionality” which prevents the exhaustive exploration of multiple local minima. For example, in the fourth system, the TM-score decreased from 0.77 to 0.30 and 0.36 with Dual Annealing and Differential Evolution (column 12 in Tables 3 and 4), whereas the performance was stable or improved with Powell and Nelder–Mead (column 12 in Tables 1 and 2).

## 4 Discussion and conclusion

In this study, we investigated the efficacy of refining AlphaFold2 predicted structures by flexibly fitting them into corresponding cryo-EM density maps using the ENM approach from ModeHunter version 1.4. The flexible fitting method utilizes normal modes derived from ENM to adjust the predicted structures and improve their agreement with the experimental maps. The new elforge.py utility finds the optimal mode elongation vector that minimizes the negative cross-correlation between the simulated map generated from the deformed structures and the target map. We tested various optimization methods, including two local methods (Powell and Nelder–Mead) and two global methods (Dual Annealing and Differential Evolution). We also explored three different sets of basis functions (modes 1–9, 7–9, and 1–12), masked and unmasked (box-cropped) density maps, and two CC similarity measures (Pearson and inner product). Our results show that the computationally inexpensive normal modes derived from ENM can play an important role in the refinement of molecular structures against experimental data, in addition to AlphaFold2 fold prediction (Section 3.1).

We used four AlphaFold2 systems with known discrepancies (screened from a larger set, see Supplementary Data) as test cases. Across these test systems, significant improvement in structure accuracy was observed when masked maps, modes 1–12, inner product, and Powell optimization were used, as evidenced by notable increases in TM-scores following flexible fitting. Although we chose only four test systems for the parameter optimization, the benefits of a larger mode range, map masking, and inner product are discussed below; thus, we do not expect them to depend on the specific test case selection (see Supplementary Data). Moreover, our findings and conclusions below are relevant not only for AlphaFold2 models but also for other flexible fitting scenarios where structure templates are fitted into larger cryo-EM maps of macromolecular assemblies.

Our results show that flexible fitting to masked maps (which in our case were masked to the true structure, and in other flexible fitting papers are carefully modeled to ensure that all density is accounted for by the fitted atomic structure) generally yields better results than raw box-cropped maps that contain densities of surrounding structures (Section 3.2). It is common practice in flexible fitting to edit maps; most tools in the community, including Situs and UCSF Chimera, provide tools for removing densities after segmentation, polygon or other shape cropping, etc. Other masking strategies, such as “Map Algebra”, can also be used based on difference mapping [Fig. 2 in Wriggers and Chacon (2001); Figs 1 and 2 in Wriggers *et al.* (2011)] to remove unaccounted for densities. Our results show that the preparation work spent on map masking pays dividends later by achieving a more accurate fit. In cases where such careful preparation is not possible, the use of box-cropped maps is better than not performing any refinement, but users need to be aware of the risk of false positive fits due to spurious densities. In such situations, non-local optimization methods, which involve a more exhaustive sampling far from the initial AlphaFold2 structure, can diminish performance and are therefore not recommended (Section 3.5).

While non-local methods, such as Dual Annealing and Differential Evolution, can theoretically escape local minima and aim for global optimization, they are often computationally expensive, and their convergence to a global optimum is not guaranteed, especially in a high-dimensional search. We saw no practical advantage of using non-local methods; therefore, we recommend the efficient Powell conjugate gradient optimizer, which was an order of magnitude faster and always among the best performing optimizers (Section 3.5).

Thus, there is one potential drawback of using a local search, namely, that the atomic structure needs to be initially well aligned with the target map density so that Powell can find the optimum fit. If necessary, an exhaustive rigid-body search can be performed prior to the flexible refinement using the Situs colores (Chacón and Wriggers 2002) utility.

A notable user-controlled parameter choice in which our results show the benefit of investing more computational resources is the range of normal modes included in the set of basis functions for the refinement. Although including higher dimensional optimization arising from modes 10–12 (Section 3.3) is computationally slower, the significant enhancement of the refined models often outweighed this efficiency penalty. We stopped at mode number 12 in our tests; however, users could explore the benefit of even higher mode numbers (that include more localized high spatial-frequency modes). Further, it is generally advisable to include rigid-body modes 1–6 in the refinement, even in cases where the initial alignment is good, because of co-dependencies among all modes during the optimization. Although there is an exponential increase in search volume associated with adding extra dimensions to the elongation vector, the use of the Powell method guarantees that high-dimensional searches are not dimensionally cursed.

Our results confirm earlier conclusions that utilizing the inner product similarity measure rooted in the Situs convention [Section 6 in Wriggers (2012)] is comparable to or better than the Pearson CC (Section 3.4). The inner product calculation in this article is simpler and faster, as it measures similarly without centering or normalizing the density maps. Further, the inner product conveniently ignores any part of the deformed structure that extends beyond the map boundaries during the fitting, as the product of these voxel values with the zero-padding values will be zero, eliminating its contribution to the final sum. This advantage simplifies the calculation process by eliminating the need for an additional zero-padding of the map before calculating the similarity.

The main advantage of our flexible fitting method is its ability to correct inaccuracies in predicted structures, particularly in regions where AlphaFold2 is poorly predicted. For instance, discrepancies in the relative positioning of secondary structure elements were effectively rectified through flexible fitting, resulting in more accurate structural models. However, one drawback observed in some cases is stretching artifacts in the refined structure (specifically with Dual Annealing and Differential Evolution optimization methods with box-cropped maps, which can yield extreme elongations; therefore, we do not recommend them; see above). This phenomenon is common with normal modes and can be easily remedied by following the ENM refinement with local structure optimizers or physics-based molecular simulation. In fact, some groups have already combined normal modes with Molecular Dynamics simulations for this purpose (Periole *et al.* 2009, Vuillemot *et al.* 2022).

Whereas our study compared Pearson CC and inner product as similarity measures between the fitted structures and target map, over the last two decades, many other developers have suggested related similarity measures [most of them CC-based; see Tables 1 and 2 in Maurer *et al.* (2024)]. Since these commonly used measures are now available in a Python library (Maurer *et al.* 2024), it is straightforward to compare them in future work. Moreover, future studies should consider exploring the benefit of using decoys as start seed points from which to initiate refinement (Alshammari *et al.* 2023). As decoy generation produces a more systematic distribution of starting elongations, it does not require inefficient and problematic non-local optimization methods, and it provides better coverage of the search space. This could potentially improve the convergence rate and the refined model accuracy when a non-local search is desired.

## Supplementary Material

vbae181_Supplementary_Data

## Data Availability

The source of ModeHunter can be freely downloaded at https://modehunter.biomachina.org in ModeHunter version 1.4.
