# Supplementary material for: Flexible fitting of AlphaFold2-predicted models to cryo-EM density maps using elastic network models: a methodical affirmation
Source: Bioinform Adv. 2024 Nov 18;5(1):vbae181. doi: 10.1093/bioadv/vbae181 (PMC11783307; doi:10.1093/bioadv/vbae181)
Supplement: vbae181_Supplementary_Data [file vbae181_supplementary_data.pdf]

Supplementary Material

for "Flexible Fitting of AlphaFold2-Predicted Models to Cryo-EM Density Maps Using Elastic Network Models: A Methodical Affirmation" by Maytha Alshammari, Jing He, and Willy Wriggers

1. Selection of AlphaFold2 Test Systems Used in The Main Paper

Table 1 shows the full set of 137 AlphaFold2 predictions, from which four test cases were ultimately selected. It also provides details for dropped candidates that could be revisited in future work. Overall, 87% of the AlphaFold2 predictions already matched the expected structures very well. Only 18 of the 137 cases exhibited a TM-score below 0.80, suggesting that these candidates (second column of Table 1) could benefit from flexible refinement against a cryo-EM map. From these candidates, four promising cases were ultimately selected after decoy generation (Alshammari et al. 2023), as described below.

Supplementary Table 1. Overview of the Data Collection Process.

All Cases[a]	Refinement Candidates[b]	Selected Cases[c]
23 cases from our previous studies	7LV9-23530-B<br>7KZZ-23093-B<br>7KU7-23035-A<br>T1047s1-D1<br>T1047s2-D3<br>7DGQ-30673-3	7KZZ-23093-B<br>T1047s1-D1<br>7DGQ-30673-3
6 cases randomly chosen from the PDB	6NAV-0397-A<br>5Y5X-6810-H	6NAV-0397-A
39 free modeling cases from CASP 15	T1129s2-D1	-
37 free modeling cases from CASP 14	T1027-D1<br>T1029-D1<br>T1040-D1<br>T1070-D1	-
32 free modeling cases from CASP 13	T0990-D2<br>T0990-D3<br>T1021s3-D1<br>T1021s3-D2<br>T1022s1-D1	-

[a]Total number of AlphaFold2 cases from the five data sources indicated in the first column.
[b]Refinement candidates exhibiting a TM-score below 0.80, indicated by PDB ID, EMD, and PDB chain ID.
[c]Cases ultimately selected in this study, as explained below.

Datasets were collected from five sources:

1) 23 cases from our previous studies of refining AlphaFold using Phenix with high, hybrid, and 4-6 Å resolution cryo-EM density maps (Alshammari et al. 2022a, Alshammari et al. 2022b).
2) 6 cases randomly selected from deposited models in the PDB that correspond to 4–10Å resolution maps. The cryo-EM density maps were downloaded from the EMDB, and the corresponding atomic structures (and amino acid sequences) were downloaded from the PDB.
3) 39 free modeling cases from CASP 15 (Kryshtafovych et al. 2023).
4) 37 free modeling cases from CASP 14 (Kryshtafovych et al. 2021).
5) 32 free modeling cases from CASP 13 (Kryshtafovych et al. 2019).

In the first dataset, 6 challenging candidates out of 23 were selected based on their AlphaFold2 model TM-score being less than 0.80. For these candidates, refinement against 5–8Å resolution maps with Phenix failed in earlier work (Alshammari et al. 2022a, Alshammari et al. 2022b). To explore the possibility of success of ENM refinement described in the main text, we systematically generated decoys for the six cases that covered the low-frequency normal modes, as described in (Alshammari et al. 2023). In this screening, we used only the first three non-trivial lowest-frequency modes (i.e., the start index was 7 and the end index was 9), setting a constant amplitude of 5Å for each mode. Each mode was sampled as a cosine wave between phase 0 and phase $+\pi$ using 10 discrete sampling steps. Only three of the six candidates were used in this study, as the other three showed no significant improvement among any of their decoys (Alshammari et al. 2023). For example, 7LV9 exhibited a prominent kink in a helix (similar to the example in Fig.4 of the main text) that made it challenging to model the sharp kink with smooth normal modes of (Alshammari et al. 2023). Choosing a wider range of modes (modes 1–12, as used in Fig. 4 of the main text) might help in this case. Another candidate we did not include was 7KU7, which presented a challenge due to an error in the placement of one of the domains. This error made it difficult for ENM to accurately reposition the domain.

In the second dataset, six cases were randomly selected from the PDB, and only two exhibited TM-scores below 0.80. 6NAV was chosen in this study due to its effective ENM refinement (see main text), whereas 5Y5X exhibited a less significant refinement success.

In the third dataset, out of 39 free modeling cases from CASP 15 (Kryshtafovych et al. 2023), only one exhibited a TM-score below 0.80 and had a cryo-EM density map available. This case was not used in this study due to its large size (608 amino acids). It is possible that a spatial decomposition would benefit this case, but it was not deemed a straightforward candidate for ENM refinement.

Additionally, 4 out of 37 free modeling cases from CASP 14 (Kryshtafovych et al. 2021) exhibited TM-scores below 0.80, but none of them had experimental cryo-EM density maps associated with them that could be used for the refinement. We recommend that interested users can create simulated maps to investigate these cases using the Situs pdb2vol tool (Alshammari et al. 2022b).

Finally, 5 candidates were selected from 32 free modeling cases from CASP 13 (Kryshtafovych et al. 2019), as they had TM-scores below 0.80 and available cryo-EM density maps. ENM decoys were generated using the protocol of (Alshammari et al. 2023) for T1021s3-D1 and T1021s3-D2. For the

other three cases, the decoys were generated with slight modifications to the ENM parameters: the mode range was set to 7–11. The amplitude was based on the RMSD between the AlphaFold2 model and the true deposited structure, and each mode was sampled using four discrete steps. These cases were excluded from the study, as no significant improvement in TM-score was observed for T1021s3-D1, T1021s3-D2, and T0990-D2 when using ENM for decoy generation. Additionally, ENM stretching artifacts were observed in some decoys of T1021s3-D2 and T0990-D3.

In summary, of the 14 low TM-score candidates that had an associated experimental cryo-EM map, 10 were excluded for various reasons, such as AlphaFold2 domain arrangement errors, sharp localized deformations, and unsuitability of the ENM motion model (at least within the decoy test framework and the parameters we used; it is possible that further explorations would be able to rescue some of these challenging candidates). Nevertheless, we believe that the four surviving systems are sufficiently diverse to support the conclusions of this paper.

2. Sequence-Dependent TM-Scores

To compare with the sequence-independent TM-scores of Tables 1-4 in the main paper, we also performed TM-align in the sequence-dependent mode. (Residue indices of systems 1 and 2 were renumbered to start from 1). The TM-scores in the following Tables are slightly smaller overall, but the relative trends remain the same as those described in the main paper: The highest improvement in prediction accuracy was achieved with the widest mode range tested (modes 1-12, including rigid body modes 1-6), using masked maps, inner product scoring, and local Powell optimization.

Supplementary Table 2. Accuracy of models before and after flexible fitting using the Powell optimization method.

System[a]	Res. (Å)[b]	AF2 TM[c]	Masked Map - Aligned AF2[d]						Boxed Map - Aligned AF2[e]					
			Pearson - Margin 10			Inner Product			Pearson - Margin 10			Inner Product		
			Modes 1–9	Modes 7–9	Modes 1–12	Modes 1–9	Modes 7–9	Modes 1–12	Modes 1–9	Modes 7–9	Modes 1–12	Modes 1-9	Modes 7–9	Modes 1-12
1	5	0.46	0.61	0.47	0.60	0.49	0.47	0.63	0.47	0.47	0.45	0.47	0.47	0.45
2	5	0.54	0.65	0.61	0.60	0.65	0.58	0.66	0.63	0.59	0.60	0.62	0.58	0.61
3	5	0.75	0.79	0.80	0.82	0.79	0.80	0.82	0.79	0.80	0.82	0.79	0.80	0.82
4	4.10	0.77	0.76	0.76	0.77	0.78	0.77	0.80	0.76	0.77	0.76	0.77	0.77	0.79

Supplementary Table 3. Accuracy of models before and after flexible fitting using the Nelder–Mead optimization method.

System[a]	Res. (Å)[b]	AF2 TM[c]	Masked Map - Aligned AF2[d]						Boxed Map - Aligned AF2[e]					
			Pearson - Margin 10			Inner Product			Pearson - Margin 10			Inner Product		
			Modes 1–9	Modes 7–9	Modes 1–12	Modes 1–9	Modes 7–9	Modes 1–12	Modes 1–9	Modes 7–9	Modes 1–12	Modes 1-9	Modes 7–9	Modes 1-12
1	5	0.46	0.47	0.47	0.46	0.47	0.47	0.47	0.47	0.47	0.47	0.47	0.47	0.47
2	5	0.54	0.59	0.61	0.60	0.60	0.58	0.59	0.56	0.59	0.59	0.59	0.58	0.58
3	5	0.75	0.80	0.80	0.82	0.79	0.80	0.82	0.79	0.80	0.81	0.78	0.80	0.81
4	4.10	0.77	0.77	0.76	0.78	0.77	0.77	0.78	0.76	0.77	0.78	0.78	0.77	0.78

Supplementary Table 4. Accuracy of models before and after flexible fitting using the Dual Annealing optimization method.

System[a]	Res. (Å)[b]	AF2 TM[c]	Masked Map - Aligned AF2[d]						Boxed Map - Aligned AF2[e]					
			Pearson - Margin 10			Inner Product			Pearson - Margin 10			Inner Product		
			Modes 1–9	Modes 7–9	Modes 1–12	Modes 1–9	Modes 7–9	Modes 1–12	Modes 1–9	Modes 7–9	Modes 1–12	Modes 1-9	Modes 7–9	Modes 1-12
1	5	0.46	0.49	0.47	0.49	0.61	0.47	0.45	0.29	0.47	0.24	0.47	0.47	0.44
2	5	0.54	0.65	0.61	0.62	0.64	0.58	0.65	0.59	0.59	0.62	0.60	0.58	0.57
3	5	0.75	0.79	0.80	0.51	0.79	0.80	0.61	0.58	0.80	0.50	0.76	0.80	0.67
4	4.10	0.77	0.80	0.76	0.80	0.80	0.77	0.80	0.38	0.77	0.36	0.80	0.77	0.73

Supplementary Table 5. Accuracy of models before and after flexible fitting using the Differential Evolution optimization method.

System[a]	Res. (Å)[b]	AF2 TM[c]	Masked Map - Aligned AF2[d]						Boxed Map - Aligned AF2[e]					
			Pearson - Margin 10			Inner Product			Pearson - Margin 10			Inner Product		
			Modes 1–9	Modes 7–9	Modes 1–12	Modes 1–9	Modes 7–9	Modes 1–12	Modes 1–9	Modes 7–9	Modes 1–12	Modes 1-9	Modes 7–9	Modes 1-12
1	5	0.46	0.61	0.47	0.61	0.61	0.47	0.45	0.39	0.47	0.15	0.56	0.47	0.44
2	5	0.54	0.64	0.61	0.62	0.65	0.60	0.62	0.63	0.59	0.61	0.63	0.58	0.60
3	5	0.75	0.79	0.80	0.82	0.79	0.80	0.82	0.79	0.80	0.48	0.79	0.80	0.82
4	4.10	0.77	0.80	0.76	0.78	0.80	0.77	0.80	0.25	0.77	0.26	0.80	0.77	0.72

[a]See Section 2.1 for details. System 1 (Lipid-preserved respiratory supercomplex), System 2 (Flagellar L-ring protein), System 3 (Cation diffusion facilitator YiiP), and System 4 (*Sulfolobus islandicus* pilus).
[b]The resolution of cryo-EM maps.
[c]The accuracy is indicated as TM-scores for models obtained from AlphaFold2.
[d]TM-scores for refined models using masked map, Pearson, inner product, and various mode ranges.
[e]TM scores for refined models using box-cropped map, Pearson, inner product, and various mode ranges.

3. References

Alshammari M, He J, and Wriggers W. 2022a. Refinement of AlphaFold2 Models against Experimental Cryo-EM Density Maps at 4-6Å Resolution. In: *2022 IEEE International Conference on Bioinformatics and Biomedicine (BIBM),* 2022a: IEEE, p. 3423-3430.

Alshammari M, He J, and Wriggers W. 2023. AlphaFold2 Model Refinement Using Structure Decoys*. Proceedings of the 14th ACM International Conference on Bioinformatics, Computational Biology, and Health Informatics*. 1-7 p.

Alshammari M, Wriggers W, Sun J, and He J. 2022b. Refinement of AlphaFold2 models against experimental and hybrid cryo-EM density maps. *QRB Discovery*: 1-23.

Kryshtafovych A, Schwede T, Topf M, Fidelis K, and Moult J. 2019. Critical assessment of methods of protein structure prediction (CASP)—Round XIII. *Proteins: Structure, Function, and Bioinformatics* 87: 1011-1020.

Kryshtafovych A, Schwede T, Topf M, Fidelis K, and Moult J. 2021. Critical assessment of methods of protein structure prediction (CASP)—Round XIV. *Proteins: Structure, Function, and Bioinformatics* 89: 1607-1617.

Kryshtafovych A, Schwede T, Topf M, Fidelis K, and Moult J. 2023. Critical assessment of methods of protein structure prediction (CASP)—Round XV. *Proteins: Structure, Function, and Bioinformatics* 91: 1539-1549.
